# Authenticity across human and synthetic influencers: a configurational review of source evaluation and social influence

**DOI:** 10.3389/fpsyg.2026.1973003

**Published:** 2026-10-15

**Authors:** Xiao-Rui Lin, Amily Fikry, Wei-Guo Zhang, Kai Ding

**Affiliations:** 1 School of Business, Shanxi Datong University, Shanxi, China; 2 Department of Entrepreneurship and Marketing, Universiti Teknologi MARA, Selangor, Malaysia; 3 School of Innovation & Entrepreneurship, Shanxi Datong University, Shanxi, China; 4 College of Management, Ningbo University of Finance and Economics, Zhejiang, China

**Keywords:** authenticity, human–AI interaction, parasocial relations, social influence, social media influencers, source credibility, trust

## Abstract

**Introduction:**

Authenticity has become a central concern in social media influencer research, yet its construction is examined differently across human, virtual, and artificial intelligence (AI)-mediated sources. This study examines how authenticity is constructed and evaluated across these influencer contexts.

**Methods:**

This study conducted a corpus-based review of 1,676 English-language research articles indexed in the Web of Science Core Collection and Scopus between July 2017 and March 2026. BERTopic modeling was employed to identify recurring semantic topics, followed by an abductive, theory-informed synthesis organized around source ontology and the dominant basis of authenticity construction. Temporal changes in relative topic prevalence were examined across the eight complete calendar years from 2018 to 2025.

**Results:**

The synthesis identified four configurations: relational human authenticity, curated human authenticity, humanized synthetic authenticity, and governed synthetic authenticity. Human-influencer research emphasizes biography, accumulated interaction, commercial congruence, and motive transparency, whereas synthetic-influencer research places greater weight on persona coherence, humanization, identity disclosure, ownership, automation, and accountability. Credibility, trust, parasocial interaction, social presence, and engagement emerge as closely associated evaluative processes. Temporal analysis revealed selective changes in relative topic prevalence, particularly concerning virtual-influencer credibility, humanization, and tourism-related applications, while most topics showed no clear linear trend.

**Discussion:**

The findings provide a corpus- and method-dependent framework for understanding how authenticity is constructed and evaluated across human and synthetic influencer contexts. The framework highlights distinct evidential foundations underlying authenticity judgments and offers a basis for further research on source evaluation and social influence.

## 1 Introduction

Influencer marketing has developed into an interdisciplinary research domain in which consumer responses, platform contexts, source credibility, and brand communication are examined through diverse theoretical and methodological perspectives (Dwivedi et al., 2021). A recent scoping review nevertheless characterizes this literature as rapidly expanding yet dispersed, complicating efforts to integrate findings across constructs and contexts (Fowler and Thomas, 2023). Within this domain, authenticity is understood not as an inherent or fixed source attribute but as an audience judgment concerning the correspondence among an influencer’s presented identity, conduct, and commercial endorsements (Zhu, 2025). Research on human social media influencers further conceptualizes perceived authenticity as multidimensional, including sincerity, expertise, uniqueness, and truthful endorsement; these dimensions may relate differently to influencer evaluations, following intentions, and purchase intentions (Lee and Eastin, 2021).

For human influencers, authenticity is relational and biographical, but it is also negotiated under commercial pressure (Nistor et al., 2025). Followers’ preference for intrinsic motivation and a noncommercial orientation can create tension when influencers enter brand partnerships (Hofstetter and Gollnhofer, 2024). To manage this tension, influencers may emphasize genuine product affinity and disclose the commercial basis of sponsored content (Chen et al., 2023). Human identity therefore does not itself guarantee perceived authenticity. Rather, audiences assess whether sponsorship choices, self-presentation, disclosure practices, and product claims remain congruent with an influencer’s established biography, domain expertise, and history of interaction with followers (Chetioui et al., 2020; Colucci and Pedroni, 2022). These studies identify biographical continuity, accumulated interaction, and commercial scrutiny as sensitizing concepts for the present synthesis, rather than prespecifying its final categories (Nistor et al., 2025).

The emergence of virtual and AI-mediated influencers further complicates this human-centered account because their identities, biographies, and communicative behaviors are designed and may be controlled by technological systems, individual creators, agencies, or brands (Gambetti and Kozinets, 2024). Their synthetic origin does not, however, make authenticity conceptually irrelevant (Zhou et al., 2026). Research involving consumers and industry experts indicates that fictional, third-party-operated virtual influencers can be evaluated through distinct manifestations of authenticity, including being true-to-ideal, true-to-fact, and true-to-self (Köles et al., 2024). The relevant evaluative question is therefore not simply whether a source is human, but whether its appearance, language, expressed values, narrative continuity, and production arrangements collectively constitute a coherent and socially intelligible persona (Audrezet et al., 2025; Yin et al., 2026).

The distinction between human and synthetic sources matters, but it does not translate into a simple hierarchy of effective and ineffective influence (Deng et al., 2024). A systematic review identifies both similarities between human and virtual influencers as advertising sources and differences in their respective limitations and capabilities (Byun and Ahn, 2023). Comparative evidence likewise suggests that source type alone may not determine whether audiences adopt an influencer’s recommendation. Instead, the underlying pathway may vary: perceived usefulness is more salient for virtual-influencer recommendations, whereas identification is more influential for human-influencer recommendations, with product type further conditioning these relationships (Belanche et al., 2024; Franke et al., 2023). Thus, recurring outcomes such as credibility, trust, engagement, and behavioral intention may arise from different combinations of evidence across source ontologies (Sands et al., 2022).

Synthetic sources also bring their production and governance arrangements into the evaluation of authenticity (Silva et al., 2025). Conceptual research distinguishes virtual influencers through customization, flexibility, ownership, and automation, attributes that differentiate them from embodied human creators (Mouritzen et al., 2024). Empirical evidence further indicates that transparency about the AI technology underlying a digital endorser can shape engagement and purchase intentions through perceived agency and experience. These relationships may also depend on audiences’ prior AI knowledge and the product context (Wang and Qiu, 2024). Technologically mediated authenticity therefore rests not only on humanlike appearance and relational behavior but also on persona coherence, source disclosure, ownership visibility, automation, human involvement, and the allocation of responsibility (Kim et al., 2025; Koning and Voorveld, 2025).

Existing reviews provide important foundations for understanding influencer evaluation. Fowler and Thomas (2023) organize influencer-marketing research by theories, contexts, constructs, and methods, identifying credibility and trust as recurring mediators and authenticity as a prominent concern in self-presentation. Byun and Ahn (2023) compare embodied human influencers with virtual or computer-generated personas through source, message, and audience dimensions. Their synthesis identifies shared relational processes alongside differences involving creator control, psychological distance, and trust, while also distinguishing the visible persona from the individuals or organizations operating it. Vila-López et al. (2025) map thematic development across a broad online-influencer corpus, tracing connections among disclosure, persuasion, identity, and source evaluation. Pujadas-Gómez et al. (2025) similarly discuss virtual influencers as both relational agents and commercial instruments. These distinctions are especially relevant as AI becomes involved in content production and interaction across both human and synthetic sources, since AI involvement does not in itself determine whether the influencer is human or digitally constructed. Where prior studies use the term “AI influencer,” the present review retains that terminology while interpreting the source according to its underlying ontology. Supplementary Table S1 compares the scope and conceptual outputs of these reviews with the present study.

These contributions establish that authenticity, credibility, and trust matter across influencer settings. A more specific question remains insufficiently organized across the four reviews: how do source ontology and the dominant basis of authenticity construction condition the relationships among these evaluations? Here, authenticity concerns the perceived coherence and sincerity of the presented identity; credibility concerns the believability of the source or message, including attributed expertise and trustworthiness; and trust concerns the audience’s willingness to rely on the source. Shared terminology can conceal differences in the evidence supporting each judgment. Personal disclosure may be interpreted through a human creator’s lived experience or through the narrative coherence of a synthetic persona. Similarly, transparency can clarify commercial motives, artificial identity, or the organization responsible for a persona. The theoretical problem concerns which relationships can be meaningfully compared across these settings and which require different assumptions about identity, experience, and responsibility. Distinguishing these evaluations is necessary to assess whether an observed difference concerns acceptance of the persona, belief in its claims, or willingness to rely on it.

This study addresses this gap by integrating research on human and synthetic influencers, including virtual and AI-mediated personas, around source ontology and the dominant basis of authenticity construction. This organization brings relational, commercial, design, and governance conditions into a common comparison while retaining authenticity, credibility, and trust as distinct evaluations. It clarifies how recurring constructs are situated within different combinations of cues and identifies relationships and boundary conditions for subsequent empirical testing. BERTopic provides the corpus-level organization supporting this synthesis; the conceptual contribution arises from interpreting the studies represented within that structure. The resulting configurations are interpretive ideal types, and the review does not establish a universal causal sequence among authenticity, credibility, and trust. This study intends to address the following research questions:

RQ1. What semantic topics characterize the literature on authenticity, trust, and credibility in human, virtual, and AI-mediated influencer marketing?

RQ2. How can the identified topics be synthesized into a theory-informed configurational framework of influencer authenticity across different source types?

RQ3. How did the relative prevalence of these topics change within the corpus from 2018 to 2025?

This study contributes a corpus-bounded framework for comparing how authenticity, credibility, and trust are constructed across different influencer settings. The four ideal types organize recurring evaluative patterns around distinct bases of judgment, including relational experience, commercial alignment, source design, and governance. This framework helps clarify why similar evaluative constructs may operate differently across human and synthetic influencers and provides a basis for developing more focused empirical questions. The study also offers practical value by identifying key considerations for influencer selection, disclosure, and campaign evaluation across different source contexts.

The remainder of the paper is organized as follows. Section 2 describes corpus construction, semantic screening, the BERTopic procedure, and topic classification. Section 3 presents the 32-topic solution, develops the four-configuration taxonomy, examines the formation and evaluation of human and synthetic authenticity, and reports the descriptive topic-prevalence analysis. Section 4 interprets the principal findings and discusses their theoretical and managerial implications, limitations, future research directions, and conclusions.

## 2 Methodology

To examine how authenticity is differentiated across human and technologically mediated influencer research, BERTopic was adopted as the primary topic-modeling approach. Topic modeling is a text-analytic technique used to identify recurring themes across a large collection of documents by grouping texts that share similar linguistic or semantic patterns. Conventional probabilistic approaches such as Latent Dirichlet Allocation rely largely on word co-occurrence and may be less effective when similar vocabulary carries different meanings across research contexts (Maier et al., 2018). BERTopic addresses this limitation by representing documents through transformer-based semantic embeddings and then combining dimensionality reduction, density-based clustering, and class-based TF-IDF to identify and describe groups of semantically related documents (Chen et al., 2026). This approach was suitable for the present review because the literature contains conceptually related but distinguishable conversations surrounding influencer authenticity, including relational and parasocial processes associated with human influencers and credibility, humanization, and source-evaluation issues associated with virtual or AI-mediated influencers. BERTopic therefore provided a systematic means of mapping these semantic distinctions across the corpus before the subsequent theory-informed synthesis.

### 2.1 Data acquisition and preprocessing

Bibliographic records were retrieved from the Web of Science Core Collection and Scopus for the period beginning in July 2017 and ending in March 2026. The starting year of 2017 was selected because preliminary scoping identified it as an early point of consolidation in social media influencer research, marked by foundational studies of influencer credibility, marketing effectiveness, and sponsorship disclosure (De Veirman et al., 2017; Djafarova and Rushworth, 2017; Evans et al., 2017). Searches were conducted across article titles, abstracts, and author keywords using a two-block Boolean strategy. The first block delineated the influencer-marketing domain, whereas the second required explicit reference to authenticity, trust, or credibility. The same Boolean search expression was used in both databases: (“influencer marketing” OR “social media influencer*” OR “digital influencer*” OR “virtual influencer*” OR “AI influencer*” OR “computer-generated influencer*”) AND (authentic* OR trust* OR credib*). Records retrieved from the two databases were merged, and bibliographic fields were standardized. Duplicates were removed primarily through DOI matching and subsequently through normalized title and publication-year matching. Eligibility was restricted to English-language research articles, and records without abstracts were excluded before semantic screening. These procedures yielded 2,020 records. This corpus represents this operational search scope rather than all research relevant to authenticity.

To improve topical precision, we applied an embedding-based relevance screen to the remaining abstracts. Each abstract and the predefined research-objective statement, “The construction and evaluation of authenticity, trust, and credibility in human, virtual, and AI-mediated influencer marketing on social media,” were encoded as dense semantic vectors using the pretrained all-MiniLM-L6-v2 SentenceTransformer model. For each record, cosine similarity was calculated between the abstract embedding and the research-objective embedding, with higher values representing closer semantic alignment with the review scope (Vossen et al., 2025). Semantic similarity has been used in previous literature-screening research to identify and prioritize potentially relevant studies (Wiwatthanasetthakarn et al., 2024). Because similarity scores are specific to the embedding model and corpus, the cutoff was determined empirically.

Records located around the prospective boundary were manually examined using their titles, author keywords, and abstracts. This examination indicated that records scoring at or above 0.45 generally addressed influencer authenticity, credibility, trust, or closely related human and synthetic source-evaluation processes, whereas records below 0.45 increasingly concerned peripheral forms of digital marketing, unrelated applications of artificial intelligence, or influencer research without a substantive connection to the focal constructs. As an additional safeguard, all 344 records that fell below the proposed threshold were manually reviewed for potential relevance. This review did not identify sufficient grounds for retaining them in the focal corpus. The threshold of 0.45 was retained as a corpus-specific operational boundary. The PRISMA 2020 framework applied in this study is visually represented in Figure 1. The final corpus comprised 1,676 articles. Figure 2 shows a sharp increase in publications, rising from 11 papers in 2017 to 547 in 2025. Both 2017 and 2026 cover partial calendar years. Figure 3 shows that the literature is distributed across multiple journals. The Journal of Retailing and Consumer Services and the Journal of Business Research were the most productive outlets, while the top 10 journals accounted for about 30.9% of the corpus.

**Figure 1:**
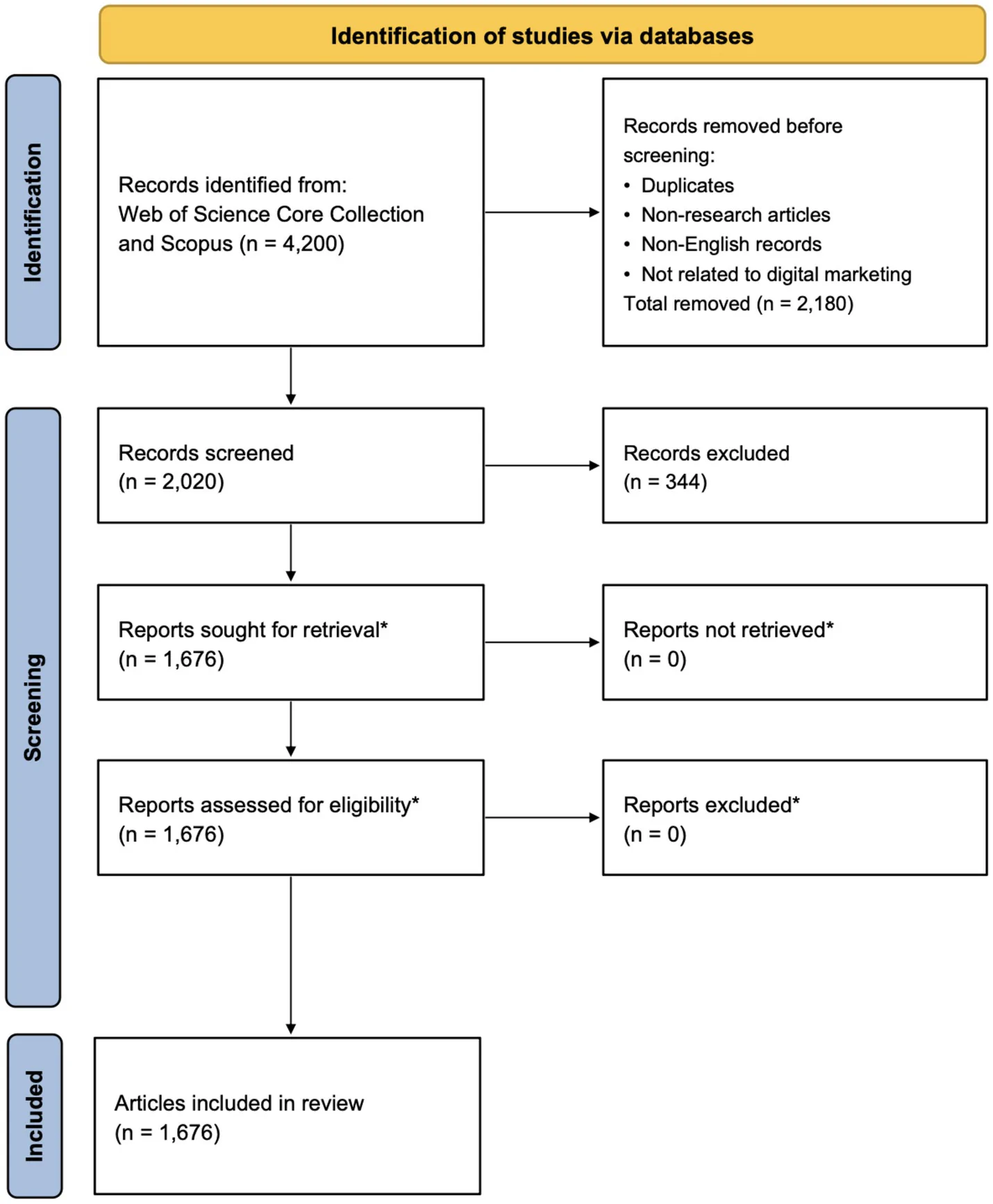
PRISMA 2020 flow diagram.

**Figure 2:**
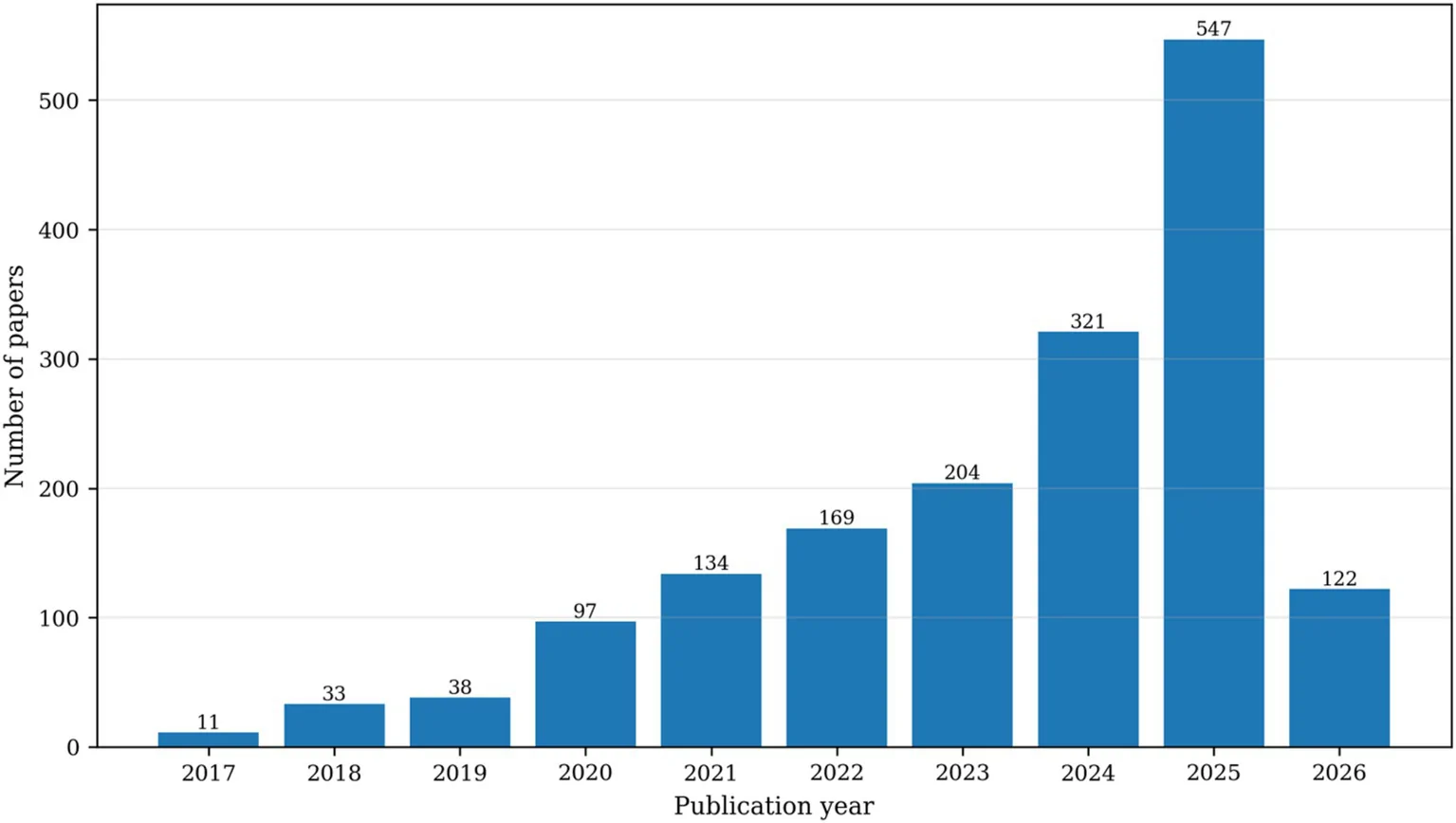
Annual distribution of publications.

**Figure 3:**
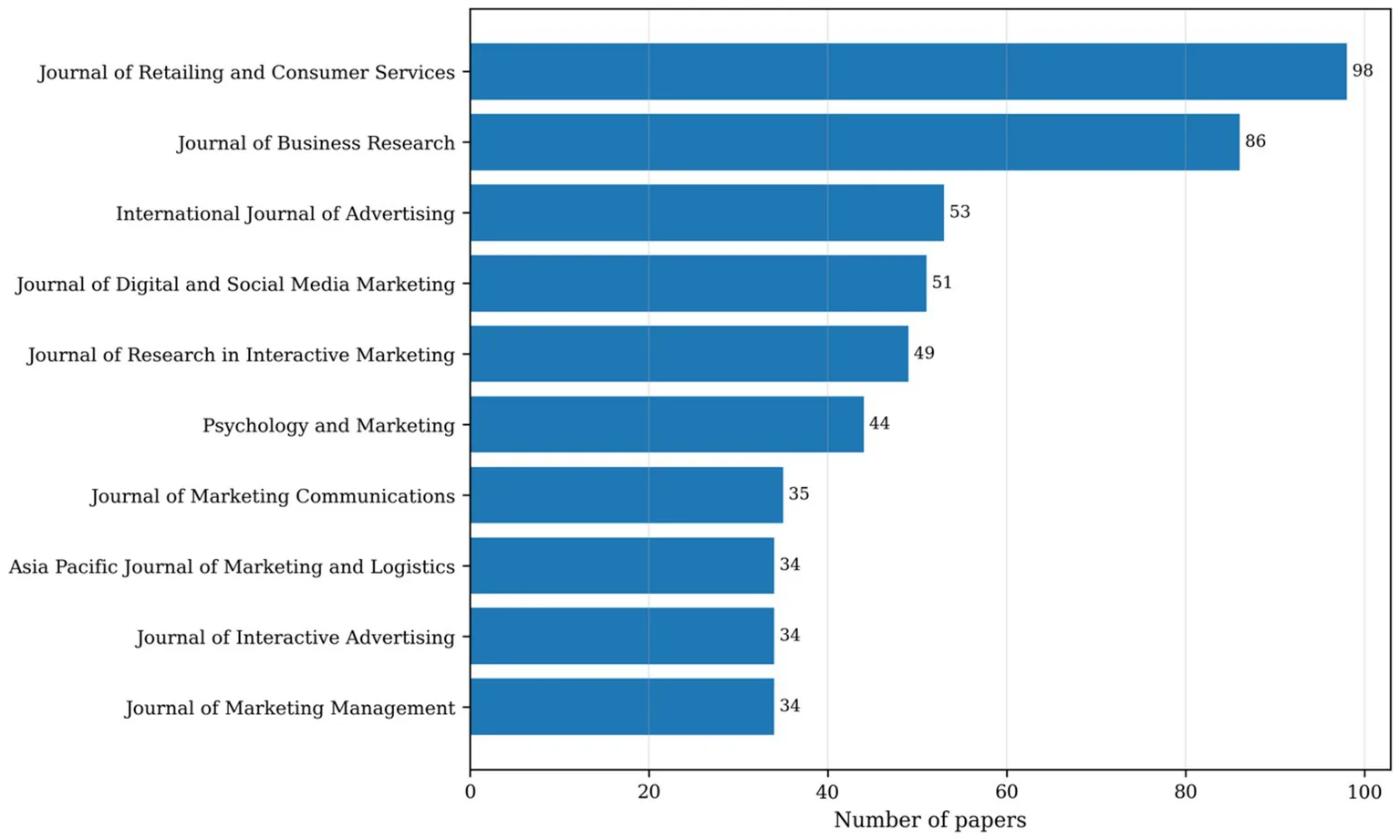
Top 10 journals by number of publications.

Consistent with BERTopic-based reviews, titles, abstracts, and available author keywords were concatenated to form the document-level modeling text (Chen et al., 2026). Only minimal cleaning, including the removal of URLs, markup, and extraneous whitespace, was applied before generating contextual embeddings. For c-TF-IDF-based topic representation, text was converted to lowercase and lemmatized, while English stopwords and noninformative characters were removed. Tokens containing fewer than three characters were excluded except for theoretically relevant abbreviations such as “AI.” The vectorizer retained unigrams and bigrams occurring in at least five documents, allowing recurrent expressions such as “parasocial interaction,” “perceived authenticity,” and “virtual influencer” to contribute to topic representation. These procedures reduced lexical noise while preserving the domain-specific distinctions required for topic interpretation (Maier et al., 2018).

### 2.2 BERTopic modeling setup

BERTopic was used to identify the semantic structure of the corpus through document embedding, dimensionality reduction, density-based clustering, and topic representation, following the general analytical procedure of Chen et al. (2026). Titles, abstracts, and available author keywords were combined into document-level texts and encoded using the pretrained all-MiniLM-L6-v2 SentenceTransformer. UMAP reduced the embeddings to five dimensions using eight nearest neighbors, cosine distance, and a fixed random seed of 42. HDBSCAN was then applied with a minimum cluster size of 20, minimum samples of 5, Euclidean distance, and the excess-of-mass cluster-selection method. Documents that did not belong sufficiently strongly to a dense cluster were assigned to Topic −1. For topic representation, CountVectorizer retained unigrams and bigrams occurring in at least five documents, and c-TF-IDF representations were further refined using KeyBERTInspired and maximal marginal relevance with a diversity parameter of 0.8. Topic probabilities were calculated for subsequent prevalence analyses.

Importantly, the four authenticity configurations were not specified as BERTopic labels, seed topics, or clustering constraints. The topic model was used first to identify corpus-level semantic groupings independently of the subsequent configurational framework. The retained solution comprised 32 topics, with 1,397 of the 1,676 articles (83.4%) assigned to these clusters and 279 articles (16.6%) assigned to Topic −1. The 32-topic solution was retained after qualitative comparison of topic coherence, distinctiveness, cluster interpretability, and alignment between topic representations and representative documents, which is treated as an exploratory semantic representation rather than an optimal or independently validated solution. Only after the topic model had been generated and interpreted were the topics compared with concepts derived from the prior literature. Source ontology and candidate authenticity cues therefore served as sensitizing concepts for the subsequent abductive synthesis rather than as inputs to BERTopic itself.

### 2.3 Topic labeling and classification

Topic interpretation and higher-order classification were conducted in two stages. First, each of the 32 substantive BERTopic topics was labeled through joint examination of its highest-weighted c-TF-IDF terms, MMR-refined terms, representative abstracts, and high-probability articles. The term representations identified the vocabulary distinguishing each cluster, while document-level inspection established how that vocabulary was used in the underlying studies. This procedure reduced reliance on isolated keywords and is consistent with recommendations that topic-model outputs be interpreted through close reading and substantive expertise (Maier et al., 2018). All researchers independently proposed preliminary topic labels. As an additional validation aid, ChatGPT 5.4 was used to assess the appropriateness of the candidate labels by reviewing the 10 most representative documents associated with each topic and comparing their dominant content with the proposed label. Its role was limited to providing a secondary consistency check and suggesting possible refinements where the label did not adequately reflect the representative documents. The final topic labels were determined and validated by the researchers through comparison of the candidate labels, model outputs, and representative documents, with disagreements resolved through consensus. The prompt and evaluation procedure used for the ChatGPT-assisted validation are provided in Supplementary Table S2.

Second, the 32 topics were examined through article-level coding to determine how they contributed to the theoretical synthesis. Representative and high-probability records were coded according to influencer type, antecedent, evaluative mechanism, outcome, moderator or contextual condition, study design, and evidential role. Based on these characteristics, topics were classified as configuration-defining, bridging, or contextual/cross-cutting. Configuration-defining topics contained a consistent combination of source characteristics, authenticity-related cues, and evaluative processes associated with one configuration. Bridging topics connected adjacent configurations by containing evidence relevant to more than one basis of authenticity construction, such as perceived authenticity across human and synthetic sources or disclosure as both a commercial and technological cue. Contextual and cross-cutting topics primarily represented industries, platforms, downstream responses, or broader consumer processes and were used to specify the contexts and outcomes surrounding the configurations rather than to define them. Table 1 presents the classification and mapping of all 32 topics and makes the evidential role of each topic explicit.

**Table 1:** Mapping of BERTopic topics to the four authenticity configurations.

Configuration	Source ontology	Dominant basis of authenticity	Primary configuration-defining topics	Secondary or bridging topics	Representative references
Relational human authenticity	Human	Biographical continuity, self-disclosure, relatability, interpersonal responsiveness, parasocial interaction, and accumulated relational experience	**T3** Source credibility in influencer marketing; **T5** Influencer attractiveness and relational formation; **T17** Platform-based credibility; **T29** Authenticity perception mechanisms	**T7** Brand–influencer authenticity links relational attachment with commercial congruence; **T16** Perceived authenticity connects human and synthetic authenticity judgments; **T20** Influencer trust dimensions contains human and virtual trust processes	Lee and Eastin (2021); Yuan and Lou (2020); Leite and Baptista (2022); Jun and Yi (2020)
Curated human authenticity	Human	Sponsorship disclosure, influencer–brand and influencer–product congruence, strategic self-presentation, content presentation, and motive transparency	**T7** Brand–influencer authenticity; **T8** Sponsorship disclosure and transparency; **T9** Celebrity and endorsement effectiveness; **T21** Brand content and engagement strategy; **T22** Visual and content cues in influencer marketing; **T23** Short-form and social media marketing	**T16** Perceived authenticity connects commercial self-presentation with broader authenticity judgments; **T8** also links commercial disclosure with governed synthetic authenticity when disclosure concerns artificial identity or AI involvement	Audrezet et al. (2020); Giuffredi-Kähr et al. (2022); Belanche et al. (2021); Janssen et al. (2022); Schouten et al. (2020)
Humanized synthetic authenticity	Virtual or AI-mediated	Anthropomorphism, social presence, emotional expression, perceived mind, behavioral realism, persona continuity, and parasociality	**T4** Human–virtual influencer comparison; **T11** Virtual influencer credibility and follower engagement; **T12** Virtual influencer engagement; **T25** Humanization of virtual influencers; **T26** Virtual influencer value creation	**T16** Perceived authenticity connects human and virtual authenticity judgments; **T20** Influencer trust dimensions contains cross-source trust processes; **T22** Visual and content cues connects designed communication with perceived warmth and authenticity; **T28** Virtual influencer design and identity construction links humanization with governance	Belanche et al. (2024); Goel and Garg (2025); Wan et al. (2024); Yoo et al. (2025)
Governed synthetic authenticity	Virtual or AI-mediated	Synthetic-identity disclosure, ownership transparency, operator control, automation, human oversight, accountability, and ethical governance	**T30** Ethics and governance of virtual influencers; **T31** Virtual influencer integration	**T8** Sponsorship disclosure and transparency extends to artificial-source disclosure; **T18** AI-assisted personalization and engagement provides adjacent evidence concerning AI transparency and human involvement; **T28** Virtual influencer design and identity construction connects visible persona design with underlying production arrangements	Köles et al. (2024); Audrezet et al. (2025); Mouritzen et al. (2024); Robinson (2020); Polyakova et al. (2025)

The higher-order organization was developed abductively by combining these topic-level patterns with established distinctions in the influencer literature. Two analytical dimensions provided the organizing structure. The first, source ontology, distinguishes embodied human influencers from virtual or AI-mediated personas. This distinction is theoretically relevant because the two source types differ in the kinds of biography, experience, agency, and authorship that audiences can attribute to them. Prior research similarly shows that human and virtual influencers can generate different evaluative processes even when they produce comparable consumer outcomes (Belanche et al., 2024). The second dimension, dominant basis of authenticity construction, identifies the primary type of evidence through which authenticity is evaluated. Across the coded topics, this evidence clustered around four recurrent foundations: relational and experiential continuity, commercial congruence and transparency, designed persona characteristics and sociality, and technological governance and accountability. These foundations are consistent with research emphasizing self-presentation, accumulated interaction, intrinsic motivation, and commercial transparency for human influencers (Audrezet et al., 2020), as well as persona coherence, humanization, disclosure, and production arrangements for virtual and AI-mediated influencers (Köles et al., 2024).

A configuration is defined as a recurring pattern in which source type, authenticity cues, and evaluative processes come together across the modeled topics and the studies represented within them. Four such patterns emerged from the synthesis. Relational human authenticity is grounded mainly in biography, interaction, relatability, and accumulated relational experience, whereas curated human authenticity places greater emphasis on commercial congruence, sponsorship, self-presentation, and transparency of motives. For synthetic sources, humanized synthetic authenticity is associated with persona design, anthropomorphism, social presence, and perceived capacity for experience, while governed synthetic authenticity draws attention to disclosure, ownership, automation, human oversight, and accountability. These configurations were developed through an abductive synthesis in which prior theory guided attention to meaningful differences in source type and authenticity-related evidence, while the modeled topics and representative studies showed how these differences were expressed across the corpus.

## 3 Results

### 3.1 Modeled topics and interpretive synthesis

For RQ1, BERTopic assigned 1,397 of the 1,676 retained articles (83.4%) to 32 topics, while 279 articles (16.6%) were assigned to Topic −1. Topic −1 is treated here as a clustering residual rather than an eligibility exclusion. Seventeen topics serve as primary configuration-defining topics in Table 1. Four additional topics (T16, T18, T20, and T28) enter the synthesis as bridging or adjacent evidence, three topics (T13, T19, and T27) are cross-cutting response topics concerning eWOM, online trust, and brand loyalty, and eight topics (T0, T1, T2, T6, T10, T14, T15, and T24) are contextual or application topics. Repeated topic IDs in Table 1 indicate conceptual links across configurations rather than duplicate clusters. This accounting makes the evidentiary sequence explicit: the 32 semantic clusters are BERTopic outputs, whereas the four configurations are a theory-informed interpretation of those outputs and the representative studies associated with them.

For RQ2, the configuration-building step organizes the modeled literature along two sensitizing dimensions: source ontology and the dominant basis on which authenticity is evaluated. Source ontology distinguishes embodied human influencers from virtual or AI-mediated personas. The second dimension distinguishes biographical and relational evidence, commercial curation, designed sociality, and production governance. Their intersection yields four overlapping ideal types: relational human authenticity, curated human authenticity, humanized synthetic authenticity, and governed synthetic authenticity. Figure 4 summarizes this interpretive organization. The configurations serve to compare the different forms of evidence through which recurring evaluations such as authenticity, credibility, and trust are discussed across source types.

**Figure 4:**
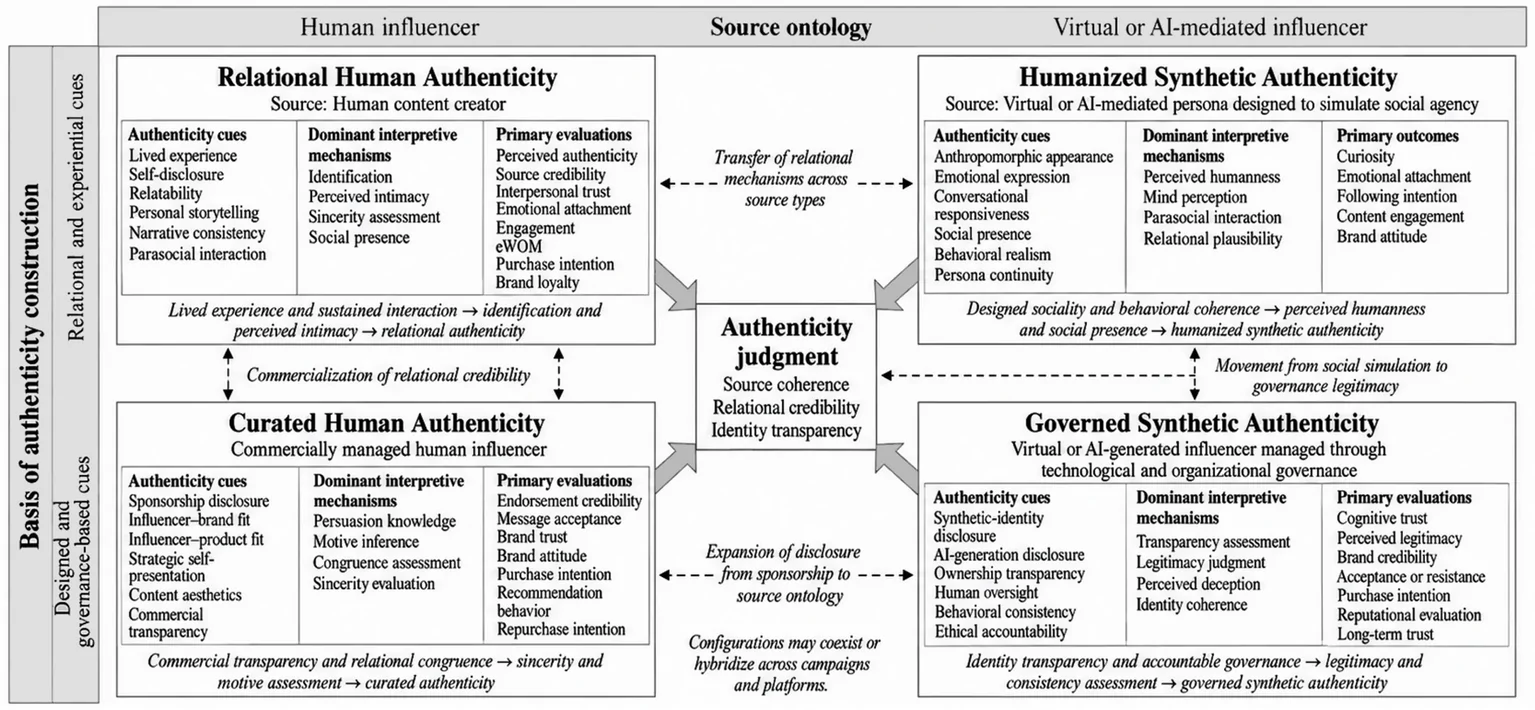
Configurational foundations of authenticity in influencer marketing.

Relational human authenticity is supported primarily by topics centered on source credibility, attractiveness and relational formation, platform-based credibility, and authenticity-perception mechanisms. Across the studies represented in these topics, authenticity is associated with the biographical plausibility of an embodied source: followers can compare self-presentation, endorsements, expertise, and interpersonal conduct with an accumulated history of observation and interaction. Perceived influencer authenticity is therefore treated as a multidimensional audience judgment rather than an inherent source property, with sincerity, truthful endorsement, expertise, uniqueness, and relational continuity contributing differently across contexts (Lee and Eastin, 2021; Wang and Weng, 2024). Interactivity, self-disclosure, and storytelling are frequently associated with stronger relational familiarity, but their contribution depends on whether these practices remain consistent with the influencer’s established identity and domain position (Leite and Baptista, 2022; Jun and Yi, 2020).

Curated human authenticity is most visible in topics concerning brand-influencer authenticity, sponsorship disclosure, endorsement effectiveness, brand content, and visual or short-form content strategy. These studies retain the importance of human biography while adding a second evaluative problem: whether a commercial relationship is compatible with the influencer’s prior identity and audience expectations. Brand fit, product congruence, motive transparency, and strategic self-presentation therefore recur as cues through which audiences interpret sponsored content. The evidence on disclosure is conditional rather than uniformly positive or negative. Disclosure may heighten persuasion knowledge and skepticism in some settings, while in others it can preserve credibility by clarifying the commercial relationship and making the recommendation easier to reconcile with the influencer’s prior conduct (Audrezet et al., 2020; Belanche et al., 2021; Xie and Feng, 2023).

Humanized synthetic authenticity draws on topics concerning human-virtual comparisons, virtual-influencer credibility and engagement, humanization, and virtual-influencer value creation. The common feature is not literal humanness but designed social intelligibility. Virtual and AI-mediated personas can display stable identities, emotion, responsiveness, and narrative continuity, allowing audiences to evaluate whether appearance, language, values, and interaction style cohere sufficiently for the persona to function as a social source. Studies of virtual-influencer authenticity, social presence, and customization show that synthetic personas can be judged through authenticity-related criteria even when their biographies are constructed, although the strength and meaning of these judgments vary with design, product category, audience expectations, and platform context (Mouritzen et al., 2024; Köles et al., 2024).

Governed synthetic authenticity is anchored most directly in topics on ethics, governance, virtual-influencer integration, and adjacent AI-assisted personalization. Here, audience evaluation can extend beyond the visible persona to the production arrangement behind it. Artificial-identity disclosure, ownership, operator visibility, automation, human oversight, and accountability become relevant because they clarify who or what is responsible for the persona’s claims and conduct. The literature does not establish a single governance effect. Technology transparency can alter engagement or purchase responses through agency and experience attributions, while other forms of disclosure can reduce perceived authenticity when audiences infer that automation has displaced human creative contribution (Wang and Qiu, 2024; Brüns and Meißner, 2024). The configuration therefore organizes a recurring governance problem rather than proposing that transparency is uniformly beneficial.

### 3.2 Human influencer authenticity

Figure 5 organizes the human-centered evidence into relational and curated foundations. Across the underlying studies, trustworthiness, expertise, informative value, similarity, familiarity, likeability, and homophily are repeatedly associated with trust, brand responses, and purchase-related outcomes, but the relative importance of these cues differs by context (Lou and Yuan, 2019; Kim and Kim, 2021). Physical attractiveness is more contingent: it can contribute to attention or parasocial evaluation in beauty, fashion, and social-media settings, yet it does not consistently predict trust or purchase intention once credibility, similarity, and relational connection are considered (Sokolova and Kefi, 2020). The human-centered evidence therefore supports a cue-configuration interpretation rather than a universal hierarchy of source attributes.

**Figure 5:**
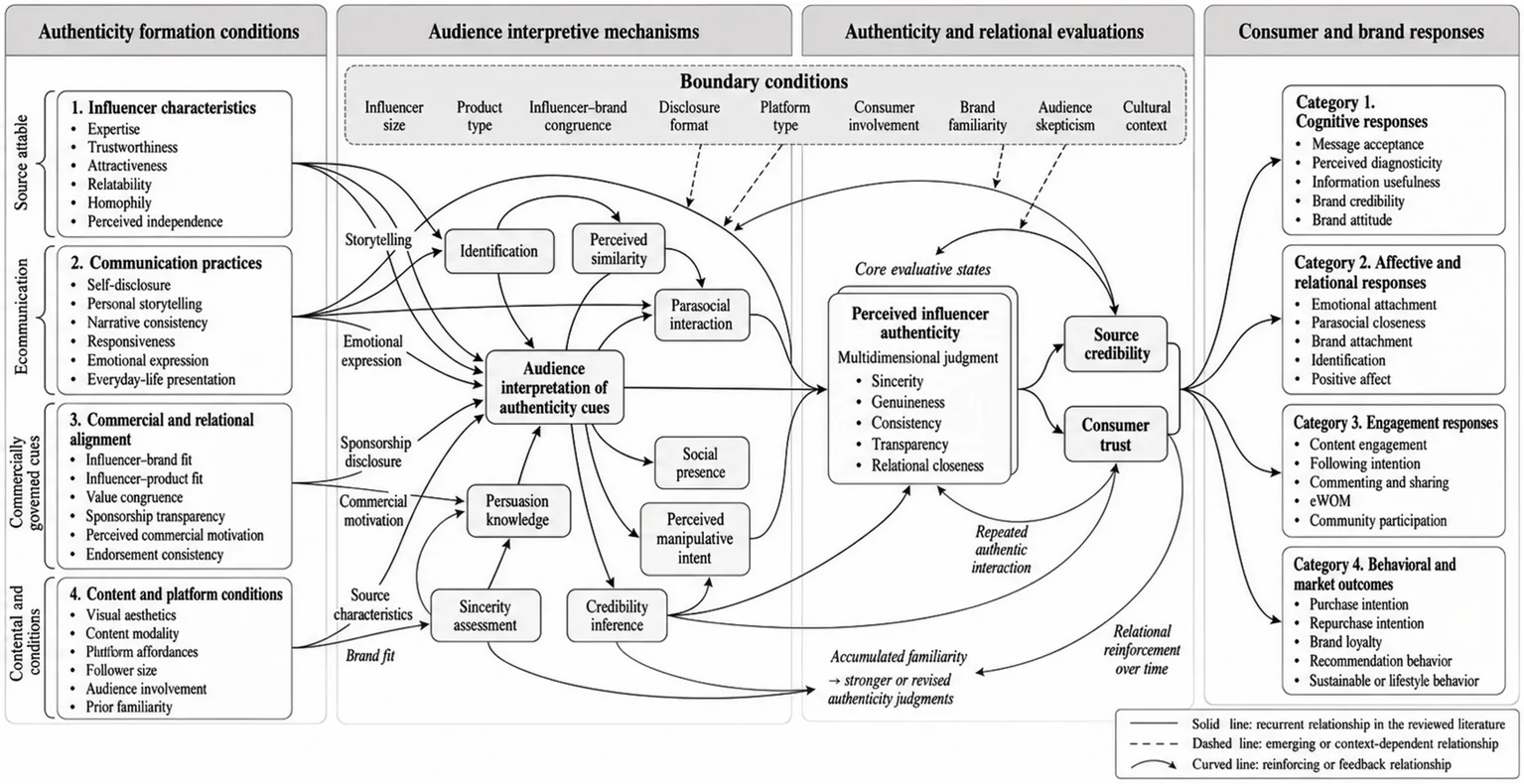
Relational and curated foundations of human influencer authenticity.

Communication practices provide the observable material through which these source characteristics are interpreted. Argument quality, information involvement, and trustworthiness are associated with credibility in YouTube influencer content (Xiao et al., 2018), while visual esthetics, content quality, interactivity, expertise, and perceived authenticity combine differently across beauty, technology, and vlogging contexts. Platform conventions further qualify the same cues. On TikTok, originality, opinion leadership, and humor are tied to following and advice adoption through platform-specific experiential mechanisms (Barta et al., 2023), whereas image-rich formats may strengthen emotional and behavioral engagement while weakening cognitive engagement under some conditions. The evidence therefore points to context-dependent relationships among source identity, message form, audience goals, and platform norms.

Commercial alignment adds another source of heterogeneity. Comparative endorsement research frequently associates perceived similarity, identification, and trust with favorable responses to influencers relative to conventional celebrities, yet product-endorser fit does not operate uniformly across product categories (Schouten et al., 2020). Campaign-level studies likewise show that expertise, hedonic content, incentives, campaign objectives, and platform type combine differently in generating engagement (Hughes et al., 2019). Within the present synthesis, curated human authenticity therefore captures the recurring need to reconcile source identity with brand, product, message, and campaign conditions rather than reducing commercial authenticity to a single fit variable.

Disclosure concentrates this evaluative problem around motive attribution. Sponsorship labels, branded-content tools, and compensation explanations can increase advertising recognition and skepticism, but their effects depend on the information supplied and the influencer’s relational history. Two-sided sponsored messages can preserve credibility by presenting the collaboration in a more balanced form (De Veirman and Hudders, 2020; Lee and Johnson, 2022). These studies suggest that audiences evaluate whether compensation, product fit, disclosure form, and message balance remain compatible with the influencer’s established identity; they do not show that disclosure has a single directional effect across contexts.

#### 3.2.1 Audience interpretation and responses to human influencer authenticity

The interpretive layer in Figure 5 groups several mechanisms reported across human-influencer studies. Perceived similarity and identification can make the source more relationally meaningful, parasocial interaction can transform repeated exposure into a felt social bond, and social presence can increase the interpersonal quality of communication. These mechanisms are frequently associated with advertising responses and purchase intention, but their relative ordering differs across models (Schouten et al., 2020; Sokolova and Kefi, 2020). More granular evidence also indicates that parasocial relationships can carry substantial direct associations while homophily, social attractiveness, and perceived influencer type condition how accessible or personally relevant the source appears (Masuda et al., 2022).

Audience interpretation is also socially situated. User comments can modify the credibility associated with parasocial relationships, indicating that influencer evaluation can be shaped by visible community responses rather than only by the follower-influencer dyad (Reinikainen et al., 2020). Social presence shows a similarly conditional role. In fitness-influencer settings, higher social presence has been associated with purchase intention and self-efficacy through parasocial interaction, whereas lower social presence can increase advertising recognition and weaken purchase intention (Kim, 2022). These findings show why persuasion knowledge and relational attachment should not be treated as mutually exclusive: followers can recognize commercial intent while still evaluating the source as credible when the interactional and contextual cues remain coherent.

Authenticity, credibility, and trust are therefore kept analytically distinct. Authenticity concerns the perceived coherence and sincerity of self-presentation; credibility concerns the believability of the source or message; trust concerns willingness to rely on the influencer over time. The empirical ordering among these evaluations varies. Two-sided communication has been linked to authenticity and credibility before downstream attitudes and behavioral intentions, whereas other studies place parasocial relationships before credibility or treat trust as a mediator of expertise, authenticity, and homophily (Lee and Johnson, 2022; Reinikainen et al., 2020; Kim and Kim, 2021). The present framework therefore organizes recurrent relationships without imposing a universal sequence among the three constructs. Additionally, downstream responses range from information processing to behavioral intention and post-purchase sharing. Desire-to-mimic research links content characteristics with perceived taste and opinion leadership before eWOM and purchase intention, while travel-decision research associates influencer trust with multiple stages of the consumer journey, including search, evaluation, purchase, satisfaction, and experience sharing (Ki and Kim, 2019; Pop et al., 2022).

### 3.3 Synthetic influencer authenticity

Figure 6 organizes the synthetic-influencer evidence around design, experiential plausibility, identity construction, and governance. The studies do not support a simple realism advantage. Humanlike virtual influencers can generate stronger engagement than more animated forms in some tourism contexts, particularly when imagery and message style make the scene easy to interpret, while other advertising research reports more favorable evaluations of human endorsers and identifies novelty or product congruence as conditions under which virtual sources become persuasive (Xie-Carson et al., 2023; Franke et al., 2023). High anthropomorphism can also produce uncanniness, with social cues moderating how humanlike design is received (Gutuleac et al., 2024). The relevant pattern is therefore conditional coherence among appearance, setting, action, and category expectations rather than increasing realism per se.

**Figure 6:**
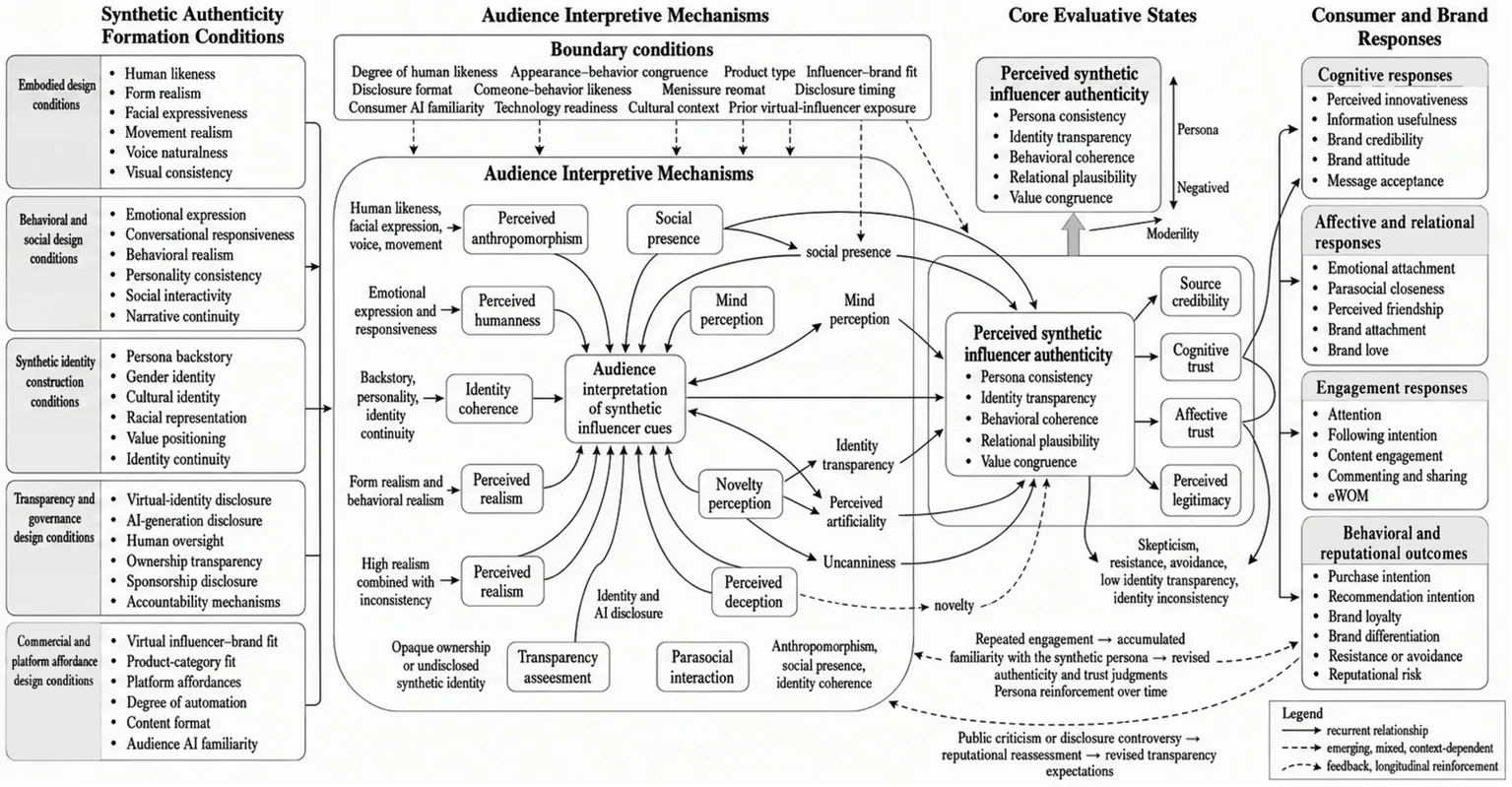
Design and identity foundations of synthetic influencer authenticity.

Perceived experiential capacity further constrains synthetic-source credibility. Consumers may attribute visual and auditory capacities to virtual influencers while doubting their ability to speak convincingly about proximal sensory experiences such as touch, smell, or taste. In these settings, purchase responses can weaken through imagery difficulty and lower perceived sensory capacity, although the disadvantage is reduced when messages emphasize distal senses or technological information that expands perceived capability (Zhou et al., 2024). Comparative studies similarly connect virtual-source status with brand attitude and purchase intention through perceived sensory capability and credibility, with product orientation and sensory-cue salience moderating the relationship (Li et al., 2023; Belanche et al., 2024). The synthesis therefore treats experiential plausibility as a boundary condition rather than an inherent deficit of synthetic sources.

Anthropomorphism is likewise heterogeneous. Humanlike appearance can strengthen warmth and competence judgments, yet other dimensions - including moral virtue, cognitive experience, emotionality, and perceived autonomy - show different relationships with credibility and parasocial outcomes (El-Hedhli et al., 2023; Dabiran et al., 2024). Consumer-experience research further shows that enjoyment, usefulness, flow, credibility, and social presence need not occupy the same mediating position across models (Kim and Park, 2024). These mixed findings justify treating anthropomorphism as a family of design cues whose effects depend on the psychological judgment under examination.

Identity construction extends beyond visual likeness. Research on computer-generated influencers locates virtual identity in relationships among followers, human influencers, brands, and the digital environment, so narrative continuity and social positioning become part of authenticity evaluation (Mrad et al., 2022). Autonomy, trustworthiness, wishful identification, and follower familiarity are also associated with parasocial relationship formation, with existing and prospective followers assigning different weights to these cues (Liu and Wang, 2025). Humanized synthetic authenticity therefore summarizes a recurring requirement for internal and relational coherence; it does not imply that all synthetic personas are evaluated through the same pathway.

#### 3.3.1 Governance and audience interpretation of synthetic authenticity

Governance becomes relevant when audiences evaluate the production arrangement behind the persona. AI-generation disclosure, sponsorship disclosure, identity declaration, ownership visibility, automation, and operator control can clarify what kind of source is speaking and who remains accountable for its claims. The evidence is mixed. Generative-AI disclosure has been associated with lower perceived brand authenticity when automation appears to replace human creation, while framing AI as assistance can reduce that penalty by preserving visible human contribution (Brüns and Meißner, 2024). In virtual-influencer advertising, realism, prosocial characteristics, and disclosure can jointly shape brand credibility, brand attitude, purchase intention, and eWOM rather than operating as independent universal effects (Phua et al., 2025).

Different disclosures also resolve different uncertainties. The visual style of the persona can alter sponsorship-disclosure effects: humanlike virtual influencers may gain through trustworthiness when sponsorship is undisclosed, whereas disclosure can remove this advantage and allow novelty to support engagement for more stylized figures (Kim et al., 2025). Origin disclosure can also reduce the psychological distance or exclusivity that supports luxury perceptions in some contexts, thereby weakening willingness to pay or purchase intention (Bie et al., 2025). Governance cues therefore carry a dual role: they can clarify accountability while also changing novelty, immersion, distance, or inferred sincerity. The synthesis preserves this heterogeneity rather than treating transparency as uniformly positive.

Audience interpretation of synthetic authenticity involves several evaluative states that should not be collapsed. Humanness, realism, social presence, mind perception, identity coherence, and perceived artificiality concern whether the persona can function as a social source. Authenticity, credibility, cognitive trust, affective trust, legitimacy, and relational acceptance are related but distinct responses. Studies of virtual-influencer outcomes similarly associate authenticity, trustworthiness, parasocial interaction, and engagement with brand loyalty, while attractiveness does not always show the same direct relationship (Vo et al., 2025). These distinctions are important because a synthetic source can be accepted as a coherent persona without being regarded as equally credible for every claim or product domain.

The downstream responses to synthetic influencers are therefore ambivalent. Novelty, controllability, scalable execution, and designed sociality can attract attention and sustain engagement, while visible fabrication, weak sensory plausibility, excessive realism, unclear disclosure, or ethically problematic representation can produce uncanniness, skepticism, resistance, or reputational risk. Consumer accounts of virtual influencers as “authentically fake” are consistent with this pattern: audiences may accept artificiality when entertainment, information, esthetics, and social integration provide value, yet weak similarity or limited parasocial connection can constrain purchase persuasion (Lou et al., 2023).

### 3.4 Temporal trends in topic prevalence

To address RQ3, we examined changes in the relative prevalence of the 32 substantive topics across the eight complete calendar years from 2018 to 2025. The partial publication years of 2017 and 2026 were excluded to improve comparability across annual observations. For each topic, annual mean topic probabilities were regressed on publication year, with the slope coefficient representing the estimated annual change in relative topic prevalence. Figure 7 presents the estimated slopes, while Supplementary Table S3 reports the complete coefficients, standard errors, 95% confidence intervals, *p*-values, and R^2^ statistics.

**Figure 7:**
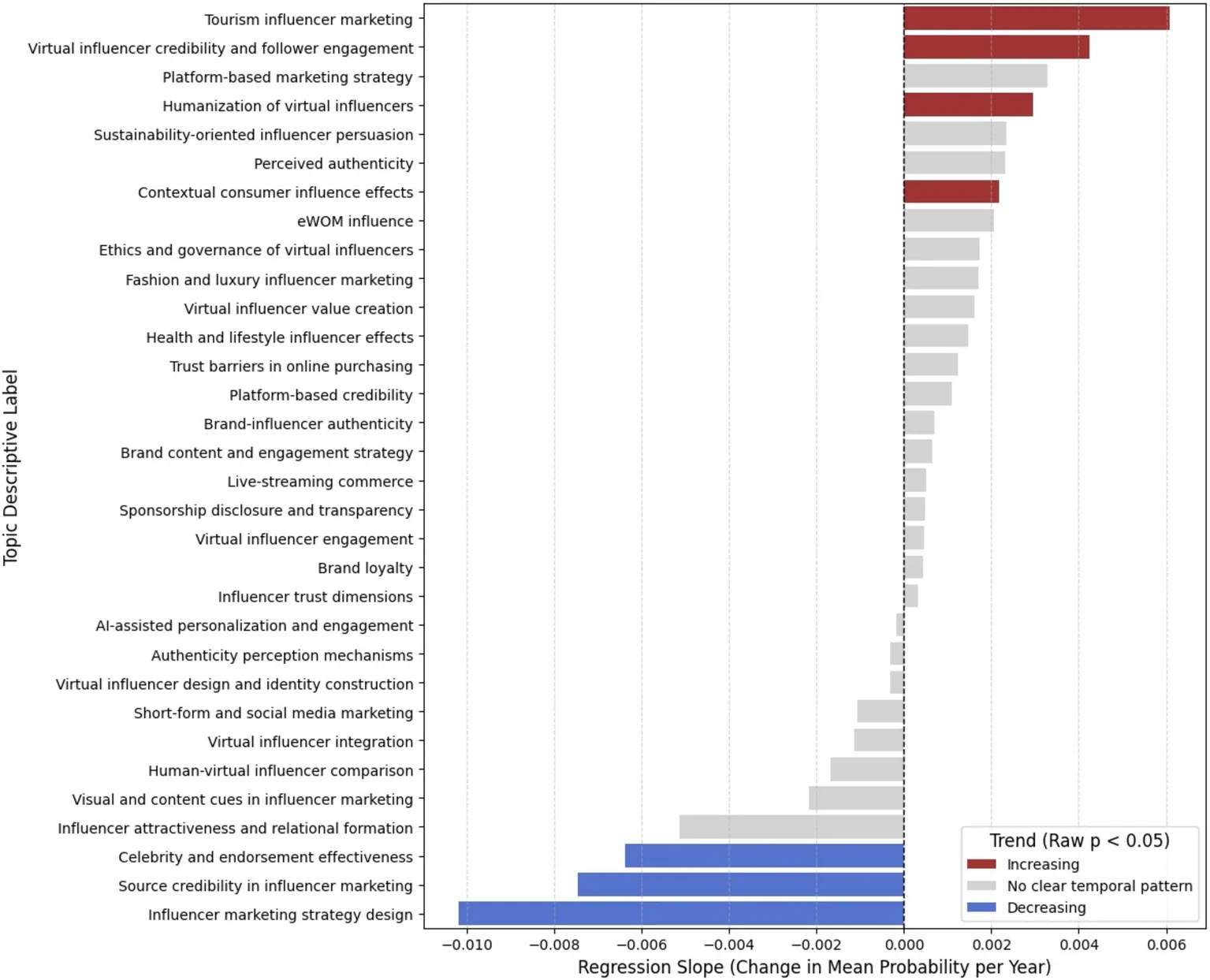
Topic trend slopes.

Figure 7 shows that tourism influencer marketing recorded the largest positive slope (b = 0.00609, 95% CI [0.00066, 0.01152], *p* = 0.033). Within synthetic-influencer research, virtual influencer credibility and follower engagement increased (b = 0.00425, 95% CI [0.00159, 0.00691], *p* = 0.008, R^2^ = 0.719), as did humanization of virtual influencers (b = 0.00296, 95% CI [0.00053, 0.00540], *p* = 0.025). Contextual consumer influence effects also increased (b = 0.00217, *p* = 0.030). These findings indicate growing relative attention to specific application contexts and the evaluative and relational properties of virtual influencers. The largest negative slope concerned influencer marketing strategy design (b = −0.01018, 95% CI [−0.01575, −0.00461], *p* = 0.004, R^2^ = 0.769). Source credibility in influencer marketing also declined (b = −0.00746, 95% CI [−0.01074, −0.00417], *p* = 0.0014, R^2^ = 0.837), alongside celebrity and endorsement effectiveness (b = −0.00639, *p* = 0.012). These decreases concern relative thematic prevalence within the corpus and do not establish declining publication counts or theoretical importance. Collectively, the results answer RQ3 by identifying selective changes in research emphasis toward particular virtual-influencer and contextual themes. Positive slopes for perceived authenticity, sponsorship disclosure, and live-streaming commerce did not reach statistical significance. The findings therefore support a qualified account of thematic differentiation.

## 4 Discussion and conclusion

### 4.1 Discussion of major findings

The semantic structure identified in this review shows that research on influencer authenticity has developed through several partially connected conversations rather than around a single conceptual core. The 32 substantive topics span source credibility, sponsorship disclosure, relational processes, virtual influencer engagement, humanization, design, and governance, while 16.6% of the corpus remained outside the substantive clusters. In relation to RQ1, this pattern suggests that authenticity, credibility, and trust are embedded in a broader set of source-evaluation processes whose emphasis differs across human and technologically mediated influencer contexts. The value of the topic map therefore lies in showing how these evaluative concerns are distributed across the literature.

This structure becomes more meaningful when the topics are considered through the four configurations developed for RQ2. Across human-influencer research, authenticity judgments are commonly connected to biography, accumulated interaction, self-disclosure, commercial congruence, and motive transparency. Relational human authenticity captures the importance of continuity between an influencer’s presented identity and accumulated audience experience, whereas curated human authenticity reflects the additional scrutiny introduced by sponsorship and commercial collaboration. This distinction helps explain why commercialization does not necessarily undermine authenticity. Brand partnerships appear to become problematic primarily when they are perceived as inconsistent with the influencer’s established identity, expertise, or prior relationship with followers (Chen et al., 2023; Hofstetter and Gollnhofer, 2024). Sponsorship disclosure is therefore better understood within this wider relational and commercial context than as an isolated transparency cue (Steils et al., 2022; Xie and Feng, 2023).

A different set of evaluative considerations becomes more visible in research on synthetic influencers. Humanized synthetic authenticity emphasizes persona coherence, anthropomorphic design, social responsiveness, and perceived experiential capacity, whereas governed synthetic authenticity shifts attention toward artificial identity, ownership, automation, human involvement, and accountability. The distinction is theoretically useful because similar outcomes, such as credibility, trust, engagement, or recommendation adoption, can be reached through different evaluative routes. Comparative research, for example, shows that perceived usefulness can be more influential for virtual-influencer recommendations, whereas identification may carry greater weight for human influencers, with product type further shaping these relationships (Belanche et al., 2024). The synthesis therefore suggests that common evaluative constructs should not automatically be assumed to rest on equivalent evidence across source types.

Transparency illustrates this distinction particularly clearly. For human influencers, disclosure generally concerns sponsorship, compensation, and commercial motives. For synthetic influencers, it can additionally reveal artificial identity, AI involvement, ownership, operator control, and the extent of human oversight. These different functions help explain why disclosure produces mixed effects across studies. AI-related transparency may strengthen perceptions of agency or accountability in some settings, while explicit AI authorship or automation may reduce perceived authenticity in others (Brüns and Meißner, 2024; Wang and Qiu, 2024). What appears to matter is not transparency in the abstract, but the particular uncertainty that the disclosure resolves and the expectations audiences hold about the source.

The four configurations also contain internal tensions that are important for interpreting the framework. Relational familiarity can strengthen credibility, but self-disclosure may become detrimental when it conflicts with perceived expertise (Leite et al., 2024). Commercial disclosure can increase persuasion awareness while remaining compatible with authenticity when the collaboration appears plausible and consistent with the influencer’s established identity (Steils et al., 2022; Xie and Feng, 2023). Similarly, anthropomorphic cues can facilitate social connection with virtual influencers while excessive realism can generate uncanniness or expose limitations in perceived sensory experience (Gutuleac et al., 2024; Zhou et al., 2024). Governance cues show a comparable tension: greater transparency can clarify agency and responsibility, but it may also make technological mediation more salient and thereby weaken authenticity judgments. These patterns indicate that authenticity-related cues acquire meaning through the interaction among source characteristics, audience expectations, and situational context.

The temporal analysis for RQ3 adds a further layer to this picture. Between 2018 and 2025, the clearest positive trends were associated with virtual influencer credibility and follower engagement, tourism influencer marketing, and the humanization of virtual influencers. By contrast, source credibility in influencer marketing, influencer marketing strategy design, and celebrity and endorsement effectiveness showed the strongest negative trends in relative prevalence. Most topics, however, did not display a clear linear pattern across the eight complete calendar years. The temporal evidence therefore points to selective changes in emphasis rather than a wholesale shift in the field. In particular, the growing representation of virtual-influencer credibility and humanization is consistent with increasing scholarly attention to technologically mediated source evaluation, while the declining relative prevalence of broader credibility and endorsement topics may reflect a gradual movement toward more context-specific research questions. These trends concern the relative composition of the analyzed corpus and should not be interpreted as evidence that earlier themes have become substantively unimportant.

### 4.2 Theoretical implications

First, this study develops a configurational account of influencer authenticity. The four configurations organize recurring evaluative constructs according to source ontology and the dominant basis on which authenticity is constructed. Relational human authenticity centers on lived experience and sustained interaction, whereas curated human authenticity incorporates commercial congruence and assessments of influencer motives. Research showing that authenticity can be co-fabricated through collaboration between influencers and brand organizations illustrates why commercial arrangements form part of this evaluation rather than merely its external context (Colucci and Pedroni, 2022). Humanized synthetic authenticity assigns greater weight to designed social cues and consistency within the digital persona. For example, virtual influencers’ humanlikeness, operator type, emotional expression, and linguistic features have been associated with different patterns of audience engagement (Ju et al., 2024). Governed synthetic authenticity, in turn, foregrounds the disclosure of artificial identity and production arrangements. The taxonomy therefore provides a basis for comparing configurations of cues without assuming that the same antecedents have equivalent meanings across source types. Because these configurations interpret semantic groupings using prior theory and selected studies, they should be treated as ideal types for further empirical examination rather than as exhaustive or mutually exclusive categories.

Second, the synthesis distinguishes continuity of constructs from equivalence of pathways. Parasocial interaction, social presence, credibility, trust, and engagement recur across human- and synthetic-influencer research, but their repeated appearance does not imply that they arise from identical cues or operate through the same processes. Comparative evidence illustrates this distinction. Belanche et al. (2024) found no overall difference between human and virtual influencers in recommendation-adoption intention, but identified different pathways involving perceived usefulness and identification, with product type further conditioning these relationships. Research on virtual influencers also shows that realism and disclosure can interact in shaping brand-related evaluations (Phua et al., 2025), while the influence of anthropomorphism may depend on racial homophily and the behavioral cost associated with the promoted action (Wan et al., 2024). Theoretical models should therefore specify the source ontology and contextual conditions under which a particular cue is expected to operate rather than treating anthropomorphism, realism, attractiveness, or disclosure as context-independent predictors.

Third, the framework clarifies the relationships among authenticity, credibility, and trust by treating them as analytically distinct evaluations. Within the framework, authenticity concerns the perceived coherence and sincerity of the source or persona; credibility concerns the believability and competence attributed to the source or message; and trust concerns an audience’s willingness to rely on that source. The reviewed evidence does not support a single ordering among these constructs. Intimate self-disclosure, for example, can reduce influencer credibility when audiences perceive the disclosed content as incongruent with the influencer’s expertise (Leite et al., 2024). Research on AI influencers similarly identifies multiple configurations associated with high trust and finds that no single antecedent is sufficient by itself (Alboqami, 2023). The framework therefore supports the examination of alternative sequences, reciprocal relationships, and context-specific configurations instead of presuming a universal mediation process.

Finally, governed synthetic authenticity shifts the analytical focus from the visible persona alone to the broader production arrangement through which that persona is created and managed. For synthetic influencers, ownership and automation are defining attributes rather than incidental implementation details (Mouritzen et al., 2024). Responsibility may also be distributed among creators, sponsoring organizations, agencies, platforms, and technology providers. Research involving multiple influencer-industry actors identifies transparency, sincerity, truthfulness, autonomy, and social responsibility as distinct ethical expectations within this organizational network (Borchers and Enke, 2022). Legal analysis further shows that influencer marketing, personalized advertising, and hybrid advertising arrangements can create regulatory ambiguities under existing digital-advertising rules (Duivenvoorde and Goantă, 2023). These studies do not establish that particular governance arrangements necessarily produce authenticity. They indicate, however, that ownership, oversight, automation, and accountability constitute theoretically relevant conditions that future research should incorporate into models of synthetic-influencer evaluation.

### 4.3 Practical implications

For brands, agencies, and human influencers, the configurational framework can serve as a diagnostic lens for campaign planning. Influencer selection should consider the joint alignment among influencer identity, content, product, platform, campaign objective, commercial arrangement, and disclosure strategy. This recommendation is consistent with evidence that functional and image fit between a brand and an influencer affect brand attitudes (Che et al., 2025). It also reflects the limitations of relying on a single visibility measure: follower count has an inverted U-shaped relationship with engagement because greater reach may be accompanied by weaker perceived relationships with followers (Wies et al., 2023). Moreover, the effects of sponsored content vary with source expertise, content characteristics, platform, and campaign intent (Hughes et al., 2019). Managers may consider assessing whether a partnership is consistent with the influencer’s established content and expertise, whether the endorsement is plausible for that source, and whether the disclosure allows audiences to interpret the commercial relationship without undermining the influencer’s prior positioning.

Organizations using virtual or AI-mediated influencers should distinguish among persona design, perceived capability, and governance. Visual humanlikeness alone should not be treated as sufficient evidence of endorsement effectiveness. Comparative research indicates that virtual influencers can generate greater perceptions of novelty even when human endorsers receive more favorable overall evaluations (Franke et al., 2023). Product assignment is particularly important when an endorsement depends on sensory experience. Consumers attribute similar visual and auditory capacities to human and virtual influencers but assign lower haptic, olfactory, and gustatory capacities to virtual personas. This difference can reduce purchase intentions for proximal-sensory endorsements, although it may be attenuated through distal-sensory framing or information about technologies that enable the relevant experience (Zhou et al., 2024). Product selection and message design should therefore reflect what a synthetic persona can plausibly perceive or experience.

Disclosure should likewise be treated as a multidimensional planning decision. In human-influencer advertising, audiences attend differently to brand tags, paid-partnership labels, and hashtags, while advertising recognition varies with disclosure, brand presence, and influencer type (Boerman and Müller, 2022). Different disclosure strategies can also produce different profiles of engagement, credibility, brand attitude, and purchase intention, making the appropriate approach dependent on the outcome being pursued (Saternus et al., 2024). Synthetic-influencer campaigns introduce additional questions concerning artificial identity, AI-generated content, ownership, degree of automation, and human oversight. Managers can pretest the format, timing, and explanation of these disclosures within the intended audience and product context. Campaign evaluation should also distinguish authenticity, credibility, trust, legitimacy, and engagement rather than combining them into a single performance measure; prior research shows that online engagement itself has been defined and measured through different affective, cognitive, and behavioral dimensions (Peltier et al., 2020). Audience comments and qualitative feedback may complement these measures by showing how users interpret the persona and its production arrangements.

### 4.4 Limitations and future research

This review has several limitations that also indicate directions for future research. The corpus was restricted to English-language journal articles indexed in the Web of Science Core Collection and Scopus and was retrieved using search terms centered on authenticity, trust, and credibility; studies framed exclusively through adjacent concepts may therefore fall outside the operational search scope. Future reviews could broaden the linguistic, database, and conceptual coverage to assess the stability of the identified topic structure. The analysis also relied mainly on titles, abstracts, and author keywords, which provide less theoretical and methodological detail than full texts; subsequent studies could integrate full-text topic modeling with qualitative synthesis or meta-analysis. In addition, the 32-topic BERTopic solution and the four authenticity configurations remain partly dependent on modeling choices and researcher interpretation, while 16.6% of articles were assigned to Topic −1. Future research could examine the robustness of these patterns in expanded corpora and empirically test the proposed configurations across human and synthetic influencer contexts. Finally, the temporal analysis was based on only eight complete annual observations from 2018 to 2025, so the observed trends should be treated as exploratory; longer-term updates could determine whether the emerging patterns persist as research on AI-mediated influencers develops.

## 5 Conclusion

This review examined how authenticity, credibility, and trust are represented across human, virtual, and AI-mediated influencer research. For RQ1, the BERTopic analysis identified 32 topics within the selected corpus, revealing a literature structured around recurring conversations on source credibility, disclosure, relational processes, virtual-influencer engagement, humanization, design, and governance. For RQ2, these topics were interpreted through an abductive synthesis that produced four configurations: relational human authenticity, curated human authenticity, humanized synthetic authenticity, and governed synthetic authenticity. Their contribution lies in distinguishing the different evidential bases through which similar evaluative constructs are formed across source types, including biography and accumulated interaction, commercial congruence, persona design, and technological governance. For RQ3, the 2018–2025 temporal analysis indicates selective changes in relative topic prevalence, with increasing attention to virtual-influencer credibility, humanization, and context-specific applications, while most topics showed no clear linear trend.

## Data Availability

The original contributions presented in the study are included in the article/Supplementary material, further inquiries can be directed to the corresponding author.
