# Supplementary material for: Authenticity across human and synthetic influencers: a configurational review of source evaluation and social influence
Source: Front Psychol. 2026 Oct 15;17:1973003. doi: 10.3389/fpsyg.2026.1973003 (PMC13716788; doi:10.3389/fpsyg.2026.1973003)
Supplement: Supplementary file 1 [file Table_1.docx]

**Supplementary material**

**Table S1. Comparison with prior reviews**

| Review | Corpus and scope | Source types | Method | Relevant constructs | Theoretical output and conclusions | Specific issue remaining |
| --- | --- | --- | --- | --- | --- | --- |
| Fowler and Thomas (2023) | 150 English-language journal articles across four databases; search window 1999–2020. Broad influencer marketing. | Primarily human social media influencers; virtual and AI influencers discussed as an emerging area. | Framework-based scoping review using theory, context, constructs/concepts, and methodology (TCCM); manual article classification. | Authenticity, credibility, trust, parasocial interaction, disclosure, and persuasion knowledge. | Maps theoretical foundations and variable roles. Identifies self-presentation, community building, and information diffusion in qualitative work; discusses tensions between authenticity and monetization. | Construct roles are catalogued without specifying when authenticity, credibility, and trust rest on shared or different evidence across human and synthetic sources. |
| Byun and Ahn (2023) | 44 publications, 2018–January 2023: 31 journal articles, 12 conference papers, and one conference review; seven search sources. | Virtual influencers compared with human-influencer findings synthesized in earlier research; a VI-focused corpus. | PRISMA-guided systematic review and quality assessment; synthesis using the revised advertising communication model of source, message, and audience. | Authenticity, credibility, trust, anthropomorphism, psychological distance, parasocial responses, and controllability. | Explains human–virtual similarities and differences, separates persona from creator and sponsor, and discusses transparency and mixed audience responses. Already questions the transferability of human-influencer findings. | Relationships among authenticity, credibility, and trust remain dispersed across communication categories. A synthesis of their distinct evidential bases and variable ordering across source types remains to be developed. |
| Pujadas-Gómez et al. (2025) | 183 WoS/Scopus publications, 2020–2024; extracted 17 December 2024, refined January–February 2025; includes non-journal formats. | Virtual and AI influencers, including comparative studies involving humans. | Bibliometric mapping; Python-based citation and collaboration analysis; thematic interpretation of metadata and abstracts. | Trust, authenticity, credibility, engagement, human-likeness, and parasocial dynamics. | Maps disciplinary and thematic concentrations, recognizes relational–commercial tensions, and calls for deeper conceptual interpretation. | Provides a VI research map rather than an explicit configurational account of authenticity, credibility, and trust across source ontologies. |
| Vila-López et al. (2025) | WoS corpus through 2021; 1,740 records reported, with inconsistent period totals and starting years. Broad online influence. | Bloggers, YouTubers, foodies, celebrities, and online endorsers; the title’s virtual-influencer label has a broad scope. | SciMAT bibliometric performance and co-word analysis; thematic evolution across three periods and strategic diagrams. | Disclosure, persuasion, credibility, expertise, identity, parasocial relations, and engagement. | Tracks shifts from blogs toward social media and from intention through disclosure to persuasion; develops an agenda organized around communication elements and application settings. | Co-word structures identify topical associations but do not distinguish the evidential bases of authenticity or their relationships with credibility and trust across human and synthetic sources. |

**Table S2. Prompt used for ChatGPT-assisted topic-label validation**

| Item | Content |
| --- | --- |
| Model | ChatGPT 5.4 |
| Purpose | To provide a secondary consistency check on researcher-generated topic labels |
| Prompt | **“You are assisting with the validation of topic labels generated from a BERTopic analysis. For the topic below, review the proposed label together with the titles and abstracts of the 10 most representative documents. Evaluate whether the proposed label accurately captures the dominant content of these documents. If the label is appropriate, state ‘Appropriate’. If refinement is needed, suggest one concise alternative label and briefly explain why. Do not create new themes that are not clearly supported by the documents.”** |

**Table S3. Topic slope statistics**

| Topic_ID | Topic_Label | Slope_Beta | Standard_Error | 95% CI | P_Value | R_Squared | Interpretation |
| --- | --- | --- | --- | --- | --- | --- | --- |
| 0 | Tourism influencer marketing | 0.00609149 | 0.00221858 | [0.00066, 0.01152] | 0.03348301 | 0.55682687 | Increasing |
| 1 | Influencer marketing strategy design | -0.0101836 | 0.00227635 | [-0.01575, -0.00461] | 0.00422087 | 0.76935191 | Decreasing |
| 2 | Platform-based marketing strategy | 0.00327616 | 0.00248951 | [-0.00282, 0.00937] | 0.23621923 | 0.22398628 | No clear temporal pattern |
| 3 | Source credibility in influencer marketing | -0.0074559 | 0.00134132 | [-0.01074, -0.00417] | 0.00143457 | 0.8373919 | Decreasing |
| 4 | Human-virtual influencer comparison | -0.0016862 | 0.00349507 | [-0.01024, 0.00687] | 0.64657613 | 0.03734564 | No clear temporal pattern |
| 5 | Influencer attractiveness and relational formation | -0.0051316 | 0.00268853 | [-0.01171, 0.00145] | 0.10489513 | 0.37779862 | No clear temporal pattern |
| 6 | Fashion and luxury influencer marketing | 0.00171797 | 0.0015408 | [-0.00205, 0.00549] | 0.30751365 | 0.1716361 | No clear temporal pattern |
| 7 | Brand-influencer authenticity | 0.00068588 | 0.00098191 | [-0.00172, 0.00309] | 0.51100311 | 0.07520634 | No clear temporal pattern |
| 8 | Sponsorship disclosure and transparency | 0.00049025 | 0.00109108 | [-0.00218, 0.00316] | 0.6689708 | 0.03255367 | No clear temporal pattern |
| 9 | Celebrity and endorsement effectiveness | -0.0063908 | 0.00181138 | [-0.01082, -0.00196] | 0.01239563 | 0.67475745 | Decreasing |
| 10 | Health and lifestyle influencer effects | 0.00147537 | 0.00160171 | [-0.00244, 0.00539] | 0.3925171 | 0.12389137 | No clear temporal pattern |
| 11 | Virtual influencer credibility and follower engagement | 0.00425096 | 0.00108586 | [0.00159, 0.00691] | 0.00784856 | 0.71865217 | Increasing |
| 12 | Virtual influencer engagement | 0.00046166 | 0.0015098 | [-0.00323, 0.00416] | 0.77010641 | 0.01534408 | No clear temporal pattern |
| 13 | eWOM influence | 0.00206036 | 0.00101866 | [-0.00043, 0.00455] | 0.08957593 | 0.40540799 | No clear temporal pattern |
| 14 | Sustainability-oriented influencer persuasion | 0.00234493 | 0.0011497 | [-0.00047, 0.00516] | 0.08749241 | 0.40944782 | No clear temporal pattern |
| 15 | Live-streaming commerce | 0.00051803 | 0.00069981 | [-0.00119, 0.00223] | 0.4870988 | 0.08368339 | No clear temporal pattern |
| 16 | Perceived authenticity | 0.00232997 | 0.00113873 | [-0.00046, 0.00512] | 0.08670755 | 0.41098932 | No clear temporal pattern |
| 17 | Platform-based credibility | 0.00109468 | 0.00172731 | [-0.00313, 0.00532] | 0.54962554 | 0.06273983 | No clear temporal pattern |
| 18 | AI-assisted personalization and engagement | -0.0001719 | 0.00122231 | [-0.00316, 0.00282] | 0.89273492 | 0.00328714 | No clear temporal pattern |
| 19 | Trust barriers in online purchasing | 0.00124699 | 0.00131334 | [-0.00197, 0.00446] | 0.37903656 | 0.13062504 | No clear temporal pattern |
| 20 | Influencer trust dimensions | 0.00032006 | 0.00142851 | [-0.00318, 0.00382] | 0.83015056 | 0.0082972 | No clear temporal pattern |
| 21 | Brand content and engagement strategy | 0.00065762 | 0.0009983 | [-0.00179, 0.00310] | 0.53451138 | 0.06744478 | No clear temporal pattern |
| 22 | Visual and content cues in influencer marketing | -0.0021712 | 0.00218658 | [-0.00752, 0.00318] | 0.35906527 | 0.14113857 | No clear temporal pattern |
| 23 | Short-form and social media marketing | -0.0010696 | 0.00125497 | [-0.00414, 0.00200] | 0.42674183 | 0.10799929 | No clear temporal pattern |
| 24 | Contextual consumer influence effects | 0.00217147 | 0.00076939 | [0.00029, 0.00405] | 0.03026112 | 0.57037205 | Increasing |
| 25 | Humanization of virtual influencers | 0.00296451 | 0.00099586 | [0.00053, 0.00540] | 0.02473766 | 0.59627375 | Increasing |
| 26 | Virtual influencer value creation | 0.0016248 | 0.00083263 | [-0.00041, 0.00366] | 0.09886723 | 0.38825151 | No clear temporal pattern |
| 27 | Brand loyalty | 0.0004299 | 0.00146259 | [-0.00315, 0.00401] | 0.77871484 | 0.01419467 | No clear temporal pattern |
| 28 | Virtual influencer design and identity construction | -0.0003265 | 0.00187798 | [-0.00492, 0.00427] | 0.86768645 | 0.00501314 | No clear temporal pattern |
| 29 | Authenticity perception mechanisms | -0.0003222 | 0.00204906 | [-0.00534, 0.00469] | 0.88021828 | 0.00410351 | No clear temporal pattern |
| 30 | Ethics and governance of virtual influencers | 0.00172866 | 0.0010468 | [-0.00083, 0.00429] | 0.14975023 | 0.31248373 | No clear temporal pattern |
| 31 | Virtual influencer integration | -0.0011313 | 0.00182736 | [-0.00560, 0.00334] | 0.55860707 | 0.06004558 | No clear temporal pattern |
